# Challenging the notion of endothelial infection by SARS-CoV-2: insights from the current scientific evidence

**DOI:** 10.3389/fimmu.2025.1443932

**Published:** 2025-02-04

**Authors:** Saravanan Subramaniam, Asha Jose, Devin Kenney, Aoife K. O’Connell, Markus Bosmann, Florian Douam, Nicholas Crossland

**Affiliations:** ^1^ Department of Pharmacology and Toxicology, Massachusetts College of Pharmacy and Health Sciences, Boston, MA, United States; ^2^ Renal Section, Department of Medicine, Chobanian & Avedisian School of Medicine, Boston University, Boston, MA, United States; ^3^ Department of Virology, Immunology and Microbiology, Boston University Chobanian & Avedisian School of Medicine, Boston, MA, United States; ^4^ National Emerging Infectious Diseases Laboratories (NEIDL), Boston University, Boston, MA, United States; ^5^ Department of Medicine, Pulmonary Center, Chobanian & Avedisian School of Medicine, Boston University, Boston, MA, United States; ^6^ Department of Pathology and Laboratory Medicine, Chobanian & Avedisian School of Medicine, Boston University, Boston, MA, United States

**Keywords:** COVID-19, endothelial cells (EC), SARS-CoV-2, coreceptors, endothelial infection

## Introduction

Coronavirus disease 2019 (COVID-19), caused by SARS-CoV-2, is a public health emergency with phenotypes ranging from asymptomatic to severe sequelae that can lead to multiple organ failure and death (1, 2). SARS-CoV-2 efficiently infects airway epithelial cells and alveolar pneumocytes, causing in high viral loads and inflammatory responses, including the interferon response (3). In hospitalized patients, COVID-19 increases the risk of venous and arterial thromboembolic events due to vascular barrier failure, edema, endotheliitis, thrombosis, and inflammatory cell infiltration (4, 5). Hypercoagulation and micro- and macro-circulatory thrombosis are major causes of multiple organ failure in COVID-19 (6). Although many people have survived COVID-19 without long-term symptoms, a considerable portion of COVID-19 survivors reportedly have continuing cardiovascular issues such as coagulopathy or bleeding disorders (7). This suggests that, in addition to the respiratory epithelium, the endothelium lining of blood vessels may also be impacted by SARS-CoV-2 infection. The pathophysiology of COVID-19 has been explored in recent reviews [reviewed in (8, 9)]. Due to the conflicting data, there is ongoing controversy about the endothelial tropism (refers to the ability of SARS-CoV-2 to interact with endothelial cells) and productive endothelial infection (viral replication within the ECs) of SARS-CoV-2. Here, we share our perspective on challenging the notion of endothelial tropism and productive endothelial infection, drawing insights from the current scientific evidence.

## Endothelial dysfunction and hypercoagulation in COVID-19

The endothelium, which lines the inside of arteries, is crucial for controlling vascular tone and preserving vascular homeostasis (10). Disseminated intravascular coagulation (DIC), vasculitis, and thrombosis can all be attributable to endothelial damage (11, 12). Numerous prevalent viruses and bacteria have been found to directly infect ECs, causing necrosis, apoptosis and/or damage to the vessel wall (13–15). Upon infection by viruses such as Dengue, Hantaan, Marburg, Lassa, and Ebola, both immune and non-immune cells (including endothelial cells, monocytes, and macrophages) express tissue factor (TF), leading to hypercoagulation and often culminating in disseminated intravascular coagulation (DIC) (16–20). Studies have demonstrated that Dengue, Ebola, and Marburg viruses can directly infect endothelial cells (ECs) and replicate within them, as reviewed in detail elsewhere (13, 21, 22). However, it remains unclear whether SARS-CoV-2 exhibits a similar phenomenon due to conflicting observations.

**Figure 1:**
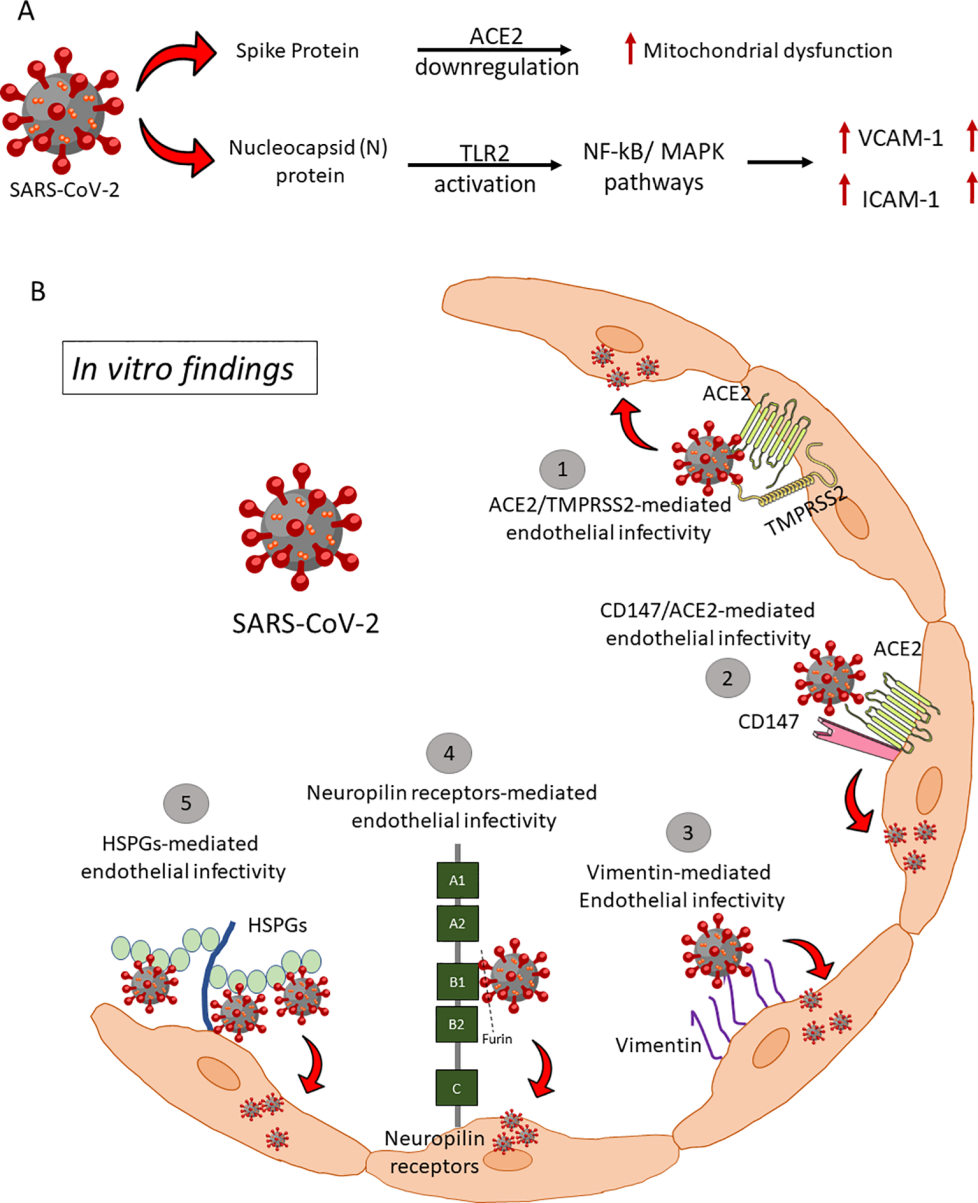
Endothelial infectivity and downstream signaling. **(A)** SARS-COV-2 Spike and Nucleocapsid proteins mediated endotheliitis and endothelial dysfunction: There are two mechanisms reported for the direct interaction of SARS-CoV-2 viral proteins with ECs and subsequent endothelial activation and dysfunction. (i) S protein of SARS-CoV-2 directly interacts with ACE2 and impairs mitochondrial function and increases redox stress which may lead to AMPK activation, MDM2 upregulation, and ultimately ACE2 destabilization. (ii) Nucleocapsid Protein (NP) of SARS-CoV-2 significantly activates human ECs through Toll-like receptor 2 (TLR2)/NF-κB and mitogen-activated protein kinase (MAPK) signaling pathways, which lead to expression of inflammatory markers (TNF-α, IL-1β) and adhesive molecules (ICAM-1, VCAM-1, and E-SELE). HSPGs, Heparan sulfate proteoglycans; ACE2, Angiotensin-Converting Enzyme 2; TMPRSS2, Transmembrane serine protease 2. **(B)** Proposed alternative (co)-receptors causing endothelial infectivity by *in vitro* assays. SARS-CoV-2 binds to Heparan sulfate and sialic acid-containing glycoproteins and gangliosides on ECs. Proteolytic cleavage of polybasic furin-type cleavage sites in the SARS-CoV-2 Spike protein was shown to expose a conserved C-terminal motif that binds cell surface Neuropilin-1 and Neuropilin-2 receptors, thus significantly potentiating SARS-CoV-2 entry, and infectivity *in vitro*. Vimentin on the endothelial surface acts as a co-receptor for SARS-CoV-2 entrance, according to two independent studies. CD147, a transmembrane glycoprotein of the immunoglobulin superfamily, has been identified as a potential receptor to bind spike protein and mediate virus entry.

SARS-CoV-2 infection of endothelium is less studied than airway epithelium and alveolar pneumocytes (10, 23–25). Despite thromboprophylaxis, 31-49% of COVID-19 emergency care patients had arterial and venous thromboembolism (26–30). This shows that endothelial impairment must be addressed aggressively to prevent thrombosis. However, it is unclear whether the hypercoagulation is driven by lung-induced systemic inflammation upon infection, or by endothelial injury or dysfunction due to direct SARS-CoV-2 infection. Several pro-inflammatory cytokines including TNF-α, IL-1α, IL-1β, IL-6, IL-8, MCP-1, IFN-γ that are responsible for the cytokine storm in COVID-19 (31, 32) may induce COVID-19-associated coagulopathy (CAC) via expression of TF on ECs, monocytes, macrophages and T cells (33–40). The IL-6 signaling complex damages liver sinusoidal ECs and produces liver injury, suggesting that endothelial dysfunction and hypercoagulation may cause severe COVID-19 (41). SARS-CoV-2 infection in the Syrian hamster model showed inflammation and type I interferon dysregulation in respiratory and non-respiratory tissues like the heart and kidney, shedding light on COVID-19 as a multiorgan disease and possible post-acute sequelae (42). SARS-CoV-2 Spike and Nucleocapsid protein directly activate ECs, inducing mitochondrial dysfunction, vasculopathy, and coagulopathy (43, 44) (**Figure 1**). Furthermore, our recent study showed that the early host response of the endothelium to SARS-CoV-2 infection declines with aging, potentially contributing to increased disease severity (45).

## Proposed novel endothelial (co)-receptors for SARS-CoV-2 entry

SARS-CoV-2 and SARS-CoV utilize human ACE2 as an entry receptor and TMPRSS2, primarily expressed by ECs in the respiratory and digestive tracts, as a co-factor to degrade extracellular matrix proteins for viral entry (46). ACE2 is variably expressed on arterial and venous ECs, smooth muscle cells, and pericytes across organs, facilitating systemic viral dissemination upon entry into the circulatory system. However, studies, including in-house immunohistochemistry, showed that humanized ACE2 mice express hACE2 in brain blood vessels but not in lung, gastrointestinal, or renal vessels, suggesting SARS-CoV-2 may employ both hACE2-dependent and independent entry mechanisms (47) (**Figure 1B**). Low expression of ACE2 and TMPRSS2 in human ECs limits the ability of SARS-CoV-2 to infect ECs (48). Thus, the variations in ACE2 expression across different microvascular beds, or alternative receptors on ECs may facilitate the entry of infectious particles. Supporting this concept, numerous additional receptors have been identified over the past three years as potentially relevant to viral particle entry in ECs. Endosomal cysteine peptidases like cathepsins B and L activate the spike (S) protein, enhancing viral entry (49–51). SARS-CoV-2 also binds heparan sulfate, sialic acid-containing glycoproteins, and gangliosides on ECs (52, 53). Proteolytic cleavage at furin-type cleavage sites in the S protein exposes a conserved motif that interacts with Neuropilin-1/2 receptors, significantly increasing infectivity (54, 55). Vimentin, CD147, and TMEM106B have been identified as co-receptors or alternative receptors, though role of TMEM106B in COVID-19 pathology lacks experimental validation (56–59). Further research is needed to confirm these mechanisms and *in vivo* relevance.

## Controversies in COVID-19 and direct infection of ECs

Endotheliitis is regarded as a host immune-inflammatory response of the endothelium forming the inner surface of blood vessels in association with a direct consequence of infectious pathogen invasion. Systemic endotheliitis causes organ damage (60). Human autopsies, non-human primates (NHPs) and mice models showed sporadic endothelial infection and consistently observed in Syrian hamsters (61, 62). Compared to healthy individuals, circulating markers of endothelial and platelet activation are elevated in severe COVID-19 (63). Evidence of myeloid polarization, such as elevated levels of shed CD16 and CD163, have been linked to the expression of TF by proinflammatory macrophages and are related with poor clinical outcomes (64).

Elevated D-dimer and thrombocytopenia in severe COVID-19 could be explained by dysregulated inflammation and microthrombus formation that are complicated by endothelial dysfunction (65). Consecutively, in patients with severe COVID-19, hypoxia due to pulmonary microvascular dysfunction might cause the classic acute respiratory distress syndrome (ARDS) (66). Furthermore, compared to controls, human pulmonary microvascular ECs isolated from human lungs challenged with lipopolysaccharide and tumor necrosis factor alpha showed increased pro-coagulant activity and PAI-1, and decreased fibrinolytic potential, emphasizing the pro-coagulant features of the pulmonary endothelium in ARDS (67).

Earlier studies support that SARS-CoV-2 viral particles were detected in highly vascularized organs in which endothelial dysfunction plays a fundamental role (24, 68–76). Initial transmission electron microscopy (TEM) studies revealed the presence of viral particles in kidney ECs, venous ECs, and liver sinusoidal ECs in autopsy samples from COVID-19 patients (76–78). However, owing to the challenges of interpreting TEM and the high variability in experience of those interpreting images, the presence of viral particle in the endothelium remains debatable (76, 79, 80). Irrespective of these controversies, there has been increasing evidence to indicate that coated vesicles and multivesicular bodies closely mimic viral particles even in the lung epithelium by TEM and are not uncommonly misinterpreted (81). Despite the thrombo-inflammatory phenotype, no definitive animal models or human biopsies have yet shown direct SARS-CoV-2 infection of ECs or the presence of viral particles (82–85). Human micro- and macrovascular ECs are resistant to SARS-CoV-2 infection, and ACE2 overexpression is necessary for endothelial infection (86–89). Montezano et al. demonstrated that recombinant Spike protein-1 induced endothelial inflammation via ACE2 independent of ACE2 enzymatic activity and viral replication *in vitro* (90). On *ex vivo* lung cultures from a patient who had SARS-CoV-2 infection found no signs of the virus in the vascular endothelium following immunohistochemical labeling of SARS-CoV-2 Spike protein (91). ECs primed with (IL-1) produced more pro-inflammatory cytokines, such as IL-6 and IL-8, and were resistant to direct SARS-CoV-2 infection (92). Furthermore, two separate investigations found that human pulmonary microvascular ECs were resistant to SARS-CoV-2 infection (MOI=0.5-3 after 2 hours of adsorption) (93, 94).

**Figure 2:**
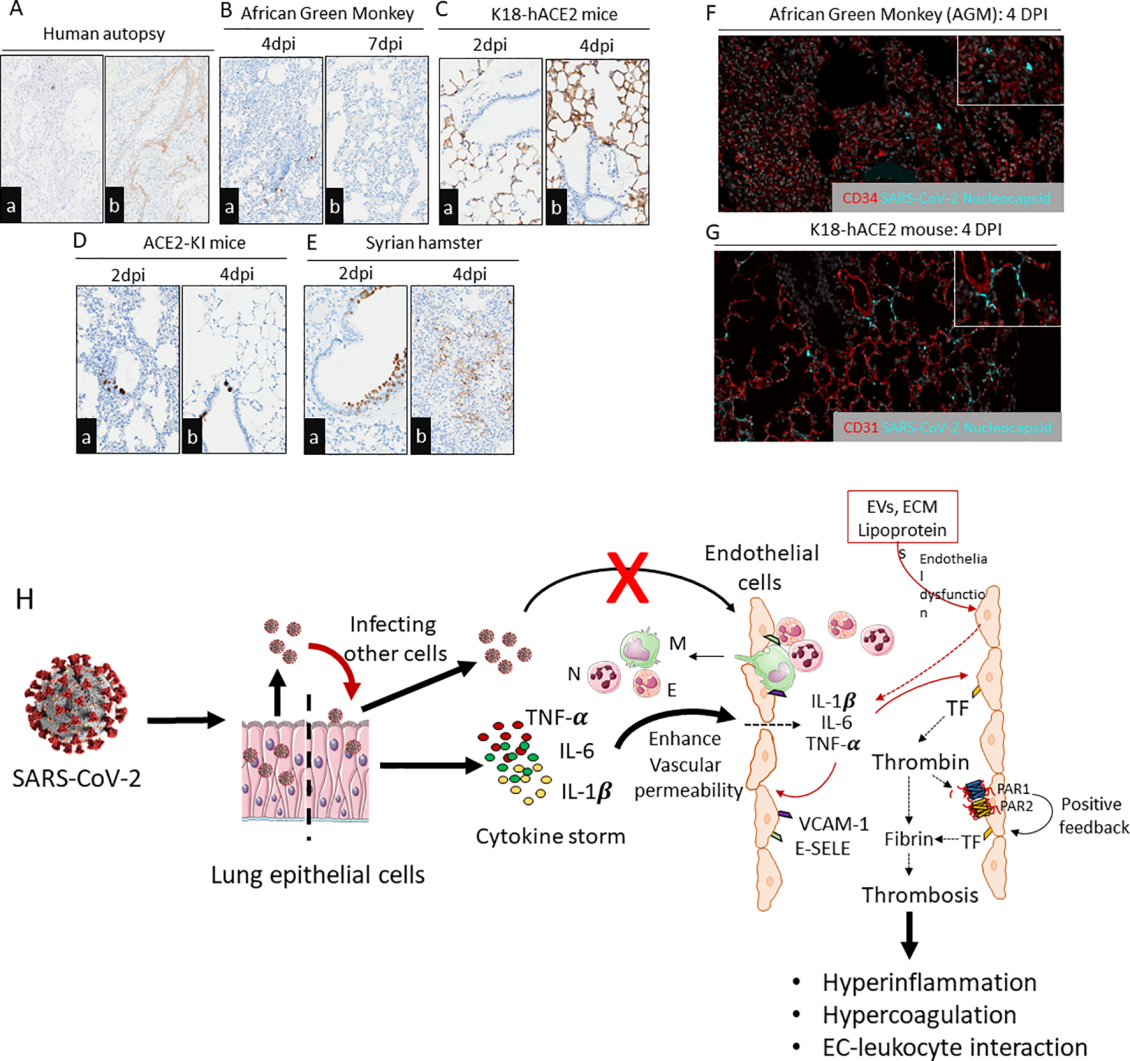
Immunohistochemistry staining of SARS-CoV-2 N protein in lung slices from different species exposed to SARS-CoV-2. **(A** ^a,b^ **)** Examination of a human autopsy illustrated absence of SARS-CoV-2 N protein in the ECs from a PCR positive patient with histologic features of diffuse alveolar damage including hyaline membranes evident by fibrinogen gamma IHC. Scale bar=200µm. **(B** ^a,b^ **)** NHPs with infected alveolar pneumocytes after 4dpi and 7dpi with no endothelial infectivity. Scale bar=200µm. **(C** ^a,b^ **)** K18-hACE2 mice infected with SARS-CoV-2 (USA/WA1/2020) illustrating N protein within alveolar pneumocytes and no endothelial infectivity. **(D** ^a,b^ **)** Global ACE2-KI mice infected with SARS-CoV-2 and N protein detectable in airways but not in ECs. Scale bar=200µm. **(E** ^a,b^ **)** Syrian hamster infected with SARS-CoV-2 with lung sections immunostained for N protein at 2dpi and 4dpi. Presence of N protein in airways was more prominent at 2dpi and alveolar pneumocytes at 4 dpi. Scale bar=200µm. **(F, G)** Absence of SARS-CoV-2 N protein localization within pulmonary endothelium of two translationally relevant preclinical models of COVID-19. **(E)** African Green Monkey, 4DPI; SARS-CoV-2 N protein localizes to alveolar pneumocytes with absence of immunoreactivity in neighboring endothelium. Red-CD34 vascular endothelial marker, Grey-DAPI, Teal-SARS-CoV-2 N protein. **(F)** K18-hACE2 mouse, 4DPI; SARS-CoV-2 N protein localizes to alveolar pneumocytes with absence of immunoreactivity of neighboring endothelium. Red-CD31 vascular endothelial marker, Grey-DAPI, Teal-SARS-CoV-2 N protein. All SARS-CoV-2 infections were from parental viruses originating from the beginning of the pandemic. All infections were from parental viruses originating from the beginning of the pandemic. Images are representative of hundreds of samples examined by a board-certified veterinary pathologist (N.A.C). **(H)** Endothelial dysfunction and endotheliitis in COVID-19- associated coagulopathy: SARS-CoV-2 infects lung epithelium and propagates within the cells for further infection. During infection, lung epithelium releases cytokines that act on endothelium and promote vascular permeability. Increased vascular permeability leads to infiltration of leukocytes into the alveolar space as well as leaking inflammatory cytokines (e.g., TNF-α, IL-6, and IL-1β) into the systemic circulation. These cytokines further stimulate luminal ECs to release more inflammatory cytokines and expression of adhesive receptors (e.g., VCAM-1, ICAM-1, E-SELE) and procoagulant receptors (e.g., Tissue factor). Elevated thrombin levels during viral infection activate the PAR1/2-dependent positive feedback mechanism, which further aggravates the expression of inflammatory cytokines, adhesive receptors, and tissue factor. These factors propagate EC-leukocyte interaction, hyperinflammation, and hypercoagulation. Other mediators like EVs, ECM, and lipoproteins trigger endothelial dysfunction which leads to expression of adhesive molecules and release of cytokines and chemokines. Direct infection of ECs by SARS-CoV-2 is an unlikely event. N, Neutrophil; M, Monocyte/macrophage; E, Eosinophil; TF, Tissue factor; PAR1, Protease-activated receptor-1; PAR2, Protease activated receptor-2; E-SELE, E-selectin; VCAM-1, Vascular cell adhesion protein 1.

Based on these findings, it is possible to hypothesize that prior reports of endothelial viral presence in patients are not a universal hallmark of illness and may be restricted to certain patient groups or isolated episodes. In light of this, we carefully evaluated the immunoreactivity of the SARS-CoV-2 Nucleocapsid (N) Protein in lung slices from translational preclinical animal models (transgenic K18-hACE2 mice (expression of hACE2 in lung epithelial cells), hACE2-KI (global hACE2 knock-in by replacing mouse ACE2), Syrian hamsters, and African green monkeys (AGM) and human postmortem lung samples. A board-certified veterinary pathologist (N.A.C.) immunohistochemically analyzed hundreds of organ slices from previous studies in each species (45, 61, 62, 69, 95–99). A PCR-positive human autopsy samples with clear hyaline membrane formation and AGMs at 7 days post-infection (dpi) showed no N Protein, suggesting viral antigen is only present during the acute phase of disease (**Figures 2A, B**). The lung epithelium of K18-hACE2, Syrian hamsters, NHPs, and ACE2-KI mice showed varying and decreasing levels of SARS-CoV-2 N protein, with airway tropism only present in ACE2-KI mice and Syrian hamsters. No tissues had viral antigens in ECs (**Figures 2C–E**). To further support the absence of direct endothelial infection, we performed duplex fluorescent IHC targeting the pulmonary endothelium (CD34 or CD31) and SARS-CoV-2 N protein. In both the AGM and K18-hACE2 mouse, luminal alveolar pneumocytes exclusively displayed SARS-CoV-2 N protein as evidenced by absence of colocalization with vascular endothelium at 4DPI (**Figures 2F, G**).

## EC and inflammatory mediators in COVID-19

Findings from our group and others collectively question the universality of direct endothelial infection by SARS-CoV-2. Because active viral infection of lung epithelial cells is associated with a systemic pro-inflammation, it is possible that inflammatory mediators can facilitate endothelial injury by activating immunothrombosis mechanisms such as complement activation, antiphospholipid antibodies, and so on. ECs treated with human sera from COVID-19 hospitalized patients (n=118) demonstrated anti-cardiolipin IgG/IgM and anti-phosphatidlyserine/prothrombin (anti-PS/PT) IgG/IgM-driven elevation of surface adhesion markers E-selectin, VCAM-1, and ICAM-1 (100–102).

Infection-induced proinflammatory cytokines, such as IL1β, TNFα, can stimulate coagulation, which may influence thrombin generation, fibrin formation, and TF-dependent thrombo-inflammatory responses via protease-activated receptors (PARs) (6, 22, 103–106) (**Figure 2H**). SARS-CoV-2 infection induces increased production of superoxide anion and release of mitochondrial DNA (mtDNA), activating Toll-like receptor 9 (TLR9) and NFκ-B. Consequently, this activation orchestrates the expression of inflammatory genes, contributing to the pathological processes associated with COVID-19 (107).

Numerous studies have highlighted changes in lipid profiles linked with COVID-19. Among the most commonly observed changes are reductions in serum cholesterol and ApoA1 levels, coupled with elevated triglycerides (108). Lipidomic analysis in COVID-19 patients showed high levels of eicosanoids in the lungs which might be a potential contributor for endothelial dysfunction.

A systemic inflammatory response and aberrant expression of the extracellular matrix (ECM) during COVID-19 controls the balance and repair of ECs (109). A study on human lung autopsy confirmed that Hyaluronan is an important compound ECM of all vital organ systems (110). COVID-19 is characterized by significantly increased levels of MMP-1 and vascular endothelial growth factor (VEGF)-A, which are directly correlated with the severity of the disease (111). More than 50% of patients who had experienced moderate or severe cases of COVID-19 had reduced pulmonary diffusion and early fibrotic changes, which were correlated with elevated levels of MMP-1 (112). A detailed dysregulation of the ECM in COVID-19 reviewed elsewhere (113).

The endothelial glycocalyx is crucial for maintaining vascular homeostasis, and it is linked to vascular endothelial dysfunction (114). Numerous studies indicate that severe COVID-19 patients experience endothelial glycocalyx damage on the endothelial cell surface, evidenced by elevated plasma levels of glycocalyx components like syndecan-1, heparan sulfate, and hyaluronan. These biomarkers, along with high levels of IL-1β, IL-6, TNF-α, hsCRP, and procalcitonin, are associated with increased severity and mortality in COVID-19 cases (115–117). Furthermore, several drugs, including heparin and tocilizumab, are already being used in COVID-19 treatment to protect the endothelial glycocalyx damage (118–121). However, despite numerous reports of glycocalyx injury in COVID-19, the underlying mechanisms are still not fully understood (117, 122–125). Vascular endothelial glycocalyx damage and potential targeted therapy in COVID-19 has been explored in recent reviews (reviewed in (84, 126, 127).

Most extracellularvesicles (EVs) found in the blood originate from platelets and erythrocytes (128). Under physiological conditions, the proportion of circulating EVs secreted by ECs is relatively low, but notably increases in pathological conditions marked by endothelial dysfunction. EVs released by ECs contain numerous endothelial markers, including endoglin/CD105, E-selectinCD62E, S-endo/CD146, vascular endothelial cadherin/CD144, platelet endothelial cell adhesion molecule 1/CD31, and intercellular adhesion molecule 1/CD54 (129). SARS-CoV-2 infection associated with the release of EVs carrying TF into the bloodstream which activate platelets and ECs, thereby contributing to COVID-19-related thrombosis in patients (130, 131).

Given the critical role of ECs in vascular homeostasis and the prospective association of COVID-19 with endothelial injury or dysfunction, it appears that patients with preexisting endothelial dysfunction in various disease states (e.g., diabetes, atherosclerosis, and hypertension) are vulnerable to a more severe disease course (132). For instance, among the comorbidities, diabetes mellitus (DM) was the most frequently reported (10.9% of cases) condition (133). Studies from China, Europe, UK and the US have also found that when people with DM acquire COVID-19, they are more likely to develop COVID-19-related complications, require ICU hospitalization, or die from the disease (134–136). The possible root cause might be chronic endothelial dysfunction due to DM together with the direct damage of ECs by SARS-CoV-2-mediated inflammatory responses result in further impairment of the microcirculation contributing to pathophysiology of acute respiratory syndrome and multi-organ failure.

## Conclusion

Experimental and clinical evidence from our group and others suggests that SARS-CoV-2 is unlikely to productively infect endothelial cells. Instead, elevated circulatory mediators, such as cytokines, extracellular matrix components, extracellular vesicles, lipids/lipoproteins, and thrombin, are likely the primary drivers of endothelial dysfunction during SARS-CoV-2 infection. Additionally, accumulating evidence indicates that SARS-CoV-2-mediated endothelial glycocalyx damage disrupts vascular homeostasis by altering vascular permeability, cell adhesion, mechanosensing, and antithrombotic and anti-inflammatory functions.

Given the mixed findings on endothelial infectivity and the involvement of multiple (co)-receptors, further research is needed. First, developing better animal models that demonstrate endothelial infectivity of SARS-CoV-2 could help identify potential co-receptors and validate direct infection. Second, there is a possibility that SARS-CoV-2 enters endothelial cells without effective replication. Initial entry may trigger significant interferon and inflammatory signaling, but rapid RNA degradation could render the infection undetectable. These hypotheses warrant further investigation.

Besides, the variation in detection of SARS-CoV-2 or viral particles might be due to the methodological differences, such as tissue sampling techniques, sensitivity of detection methods, and variations in experimental models. In addition, biological variability, including differences in patient population and disease severity, could lay a role in the observed variability across studies. Thus, further systematic review is required to identify the methodological approaches contributes to discrepancies.

Alternatively, infected ECs can be cleared from the system by various immune mechanisms, such as phagocytosis by immune cells like macrophages, or through apoptosis induced by immune responses. This can make it challenging to detect infections or inflammation in the endothelial layer, as the infected cells may be removed before they can be adequately studied or identified. However, considering that studies typically sample at different time points, the likelihood of this occurring is low. Nonetheless, it is important to carefully investigate this possibility to draw a well-informed conclusion, ensuring that immune clearance mechanisms are accounted for in the analysis.Nonetheless, current clinical and experimental evidence confirms that endothelial dysfunction is a hallmark of COVID-19, making it a critical therapeutic target. Addressing this dysfunction may help protect vulnerable individuals from severe hyperinflammation and hypercoagulation. To mitigate pro-thrombotic complications, the International Society on Thrombosis and Haemostasis (ISTH) recommends universal standard thromboprophylaxis with low molecular weight heparin or unfractionated heparin (LMWH/UFH) for all hospitalized patients, unless contraindicated (137).
